# Deep ensemble learning and transfer learning methods for classification of senescent cells from nonlinear optical microscopy images

**DOI:** 10.3389/fchem.2023.1213981

**Published:** 2023-06-23

**Authors:** Salvatore Sorrentino, Francesco Manetti, Arianna Bresci, Federico Vernuccio, Chiara Ceconello, Silvia Ghislanzoni, Italia Bongarzone, Renzo Vanna, Giulio Cerullo, Dario Polli

**Affiliations:** ^1^ Department of Physics, Politecnico di Milano, Milan, Italy; ^2^ Department of Advanced Diagnostics, Fondazione IRCCS Istituto Nazionale dei Tumori Milano, Milan, Italy; ^3^ CNR-Institute for Photonics and Nanotechnologies (CNR-IFN), Milan, Italy

**Keywords:** deep learning, transfer learning, ensemble learning, machine learning, neural networks, therapy-induced senescence, non-linear microscopy, multimodal imaging

## Abstract

The success of chemotherapy and radiotherapy anti-cancer treatments can result in tumor suppression or senescence induction. Senescence was previously considered a favorable therapeutic outcome, until recent advancements in oncology research evidenced senescence as one of the culprits of cancer recurrence. Its detection requires multiple assays, and nonlinear optical (NLO) microscopy provides a solution for fast, non-invasive, and label-free detection of therapy-induced senescent cells. Here, we develop several deep learning architectures to perform binary classification between senescent and proliferating human cancer cells using NLO microscopy images and we compare their performances. As a result of our work, we demonstrate that the most performing approach is the one based on an ensemble classifier, that uses seven different pre-trained classification networks, taken from literature, with the addition of fully connected layers on top of their architectures. This approach achieves a classification accuracy of over 90%, showing the possibility of building an automatic, unbiased senescent cells image classifier starting from multimodal NLO microscopy data. Our results open the way to a deeper investigation of senescence classification via deep learning techniques with a potential application in clinical diagnosis.

## 1 Introduction

Cancer is a complex and heterogeneous disease characterized by uncontrolled cell growth, invasion, and metastasis. While great strides have been made in cancer diagnosis and treatment, recurrence remains a major concern (Sempokuya et al., 2019). One emerging and critical area of research is the study of therapy-induced senescence (TIS) in cancer cells. TIS is a state of permanent growth arrest induced by various cancer treatments, including chemotherapy and radiotherapy. Nonetheless, TIS cells maintain metabolic activity and can contribute to disease recurrence through various mechanisms. In fact, senescence does not only affect the events inside the cell but has also the potential to affect its surrounding micro-environment. Senescent cells can communicate with neighboring cells by secreting growth factors and inflammatory cytokines, which can alter the behavior of nearby non-senescent cells promoting cancer development, with detrimental effects on the health of the organism (Kahlem et al., 2004). Therefore, it is essential to accurately identify TIS cells to prevent cancer recurrence. Cellular senescence manifests itself in response to cellular stressors as a collection of heterogeneous but overlapping phenotypes. As such, there is no marker which can identify it with absolute specificity, so that multiple, consecutive assays are usually required (Gorgoulis et al., 2019). Currently, the gold standard for detecting TIS cells relies on laborious and time-consuming techniques such as senescence-associated beta-galactosidase (SA-β-Gal) staining, gene expression and morphology analysis (Gorgoulis et al., 2019).

In this context, noninvasive, quantitative, label-free optical techniques could offer a desirable alternative. As an example, the Jones group studied the autofluorescence emission from endogenous lipopigments of replicative senescent cells using flow cytometry methods (Zhai et al., 2021). The results showed that replicative senescent cells yielded a higher fluorescence signal with respect to normal human mesenchymal stem cells. However, fluorescence signals can sometimes be nonspecific and usually require exogenous staining with fluorophores that can affect the viability of biological samples. Furthermore, they often require complex preparation treatments to ensure proper binding with their biological target, leading to toxic results over time (Liu et al., 1999). Finally, excitation using short wavelengths can induce photobleaching of fluorophores and damage to the sample, e.g., through the generation of reactive oxygen species, and limit the penetration depth in thick samples.

A solution comes from Raman scattering microscopy, a powerful imaging technique that provides information about the molecular composition of biological samples in a label-free and noninvasive way. The technique is based on the inelastic scattering of photons by molecules, which generates a unique vibrational spactrum that is characteristic of the molecular structure. Raman scattering microscopy can be used to study a wide range of biological samples, including cells, tissues, and biomaterials, and can provide valuable insights into their chemical composition, molecular structure, and biochemical processes. In prior studies, Eberhardt et al. (2017), Bai et al. (2015) utilized spontaneous Raman to identify senescence-associated biomolecular changes in senescent human fibroblasts and mesenchymal stem cells, respectively. Specifically, they observed decreased levels of nucleic acids and proteins, and increased levels of lipids, and discovered that the ratio of Raman peaks associated with protein vibrations could serve as a marker for the senescent phenotype. However, these studies focused on replicative senescence and did not investigate the critical phenotype of TIS, which is crucial for understanding the risk of cancer recurrence and resistance to treatment. To address this, Mariani et al. (2010) studied TIS induced by oxytetracycline treatment in MCF-7/NeuT human breast cancer cells with spontaneous Raman microscopy. The group observed spectral differences in the nuclei of senescent cells, suggesting nuclear membrane instability as a key feature of TIS. On a different note, Oh et al. (2022) used quantitative Stimulated Raman Scattering (SRS) to investigate cytoplasmic concentration changes during cellular senescence. Compared to spontaneous Raman, SRS coherent signal generation greatly amplifies the Raman scattering processes, allowing for faster imaging speed, higher chemical sensitivity, and finer spatial resolution (Cheng et al., 2022). Moreover, SRS microscopy possesses intrinsic 3D optical sectioning capabilities and can be seamlessly coupled with other Nonlinear Optical (NLO) modalities, such as Two-Photon Excited Fluorescence (TPEF), also requiring ultrashort laser pulses in the red or near-infrared spectral range, for multimodal imaging (Lu et al., 2015; Talone et al., 2022). The study by Oh *et al.* associates lipids and proteins upregulation to the TIS cells phenotype, but lacks any time resolution, since they investigated only one time point and no measurements were conducted during the first hours after treatment, at the onset of senescence. In our previous work (Bresci et al., 2023), we successfully identified quantitative markers of TIS *in vitro* in human hepatic cancer cells, treated with a chemotherapeutic drug, via label-free multimodal NLO microscopy combining SRS and TPEF. We studied the onset and progression of TIS at consecutive time-points, up to 7-days post treatment, finding quantitative metabolic and biochemical indicators. We observed that these markers can spot early-stage senescence induction as early as 24 h after therapy in unperturbed culture conditions, making label-free NLO microscopy a perspective candidate for TIS detection in clinical practice.

However, all the presented research works share a common feature, that is, they rely solely on statistical evaluations, which can be biased by the choice of the model and provide only a narrow picture of the multifaceted TIS phenotype. Traditional statistical methods have low uncertainty tolerance and rely on *a priori* assumptions, such as the type of error distribution and the additivity of the parameters within the linear predictor, which are often not met in clinical practice and may be overlooked in the scientific literature. In contrast, deep learning techniques are free from such assumptions and offer greater flexibility. Deep learning methods can take advantage of all the available information in a dataset, providing a more comprehensive understanding of the underlying patterns and relationships (Rajula et al., 2020).

In recent years, deep learning has been proven as an extremely useful tool for image classification in the biomedical field. In the context of deep learning, the classification task is usually performed using an Artificial Neural Network (ANN) in a supervised learning approach (Alzubaidi et al., 2021). ANN are composed of layers of artificial neurons connected with each other and, starting from the data in input, they can predict at the output the class to which the input belongs. Layers which are not connected to the input or output of the ANN are called hidden layers. Based on the number of hidden layers, ANNs can be classified either as shallow (one or two hidden layers) or deep (more than two hidden layers). For image classification, due to the complexity of the input data, in 1998 Lecun et al. (1998) proposed a new class of ANN, the so-called Convolutional Neural Network (CNN), which can perform image classification using far less parameters than a usual ANN, reducing the training time and improving the performances (Vernuccio et al., 2022).

ANNs based on CNNs have shown great potential in cancer diagnosis, including the identification of cancer subtypes, prediction of treatment response, and detection of metastatic lesions. Recent studies have demonstrated the potential of deep learning in the diagnosis of various types of cancer, including breast cancer, lung cancer, and skin cancer (Dildar et al., 2021; Mridha et al., 2021; Wang, 2022). For example, CNN-trained systems have been developed to accurately detect breast cancer masses from mammography images, with performance comparable to that of expert pathologists (Kooi et al., 2017). Similarly, deep learning models have been used to classify skin lesions and predict melanoma with high accuracy, potentially allowing for earlier detection and improved patient outcomes (Mahbod et al., 2019).

However, there are still several obstacles that need to be addressed in the development and application of deep-learning methods for cancer diagnosis. One of the major challenges is the availability of large, high-quality datasets that are representative of the diverse range of cancer cell types and stages. Additionally, there is a need to develop interpretable models that can provide insight into the features and biomarkers that contribute to accurate cancer classification (Teo et al., 2021). While deep-learning models can achieve high levels of accuracy in cancer diagnosis, they are often considered “black boxes” in that it can be difficult to understand how the model arrived at its predictions, namely which are the principal biomarkers that stimulate the neural network towards successful classification. This lack of interpretability can be a barrier to clinical adoption, as physicians may be hesitant to rely on a model whose inner workings they do not fully understand. As such, there is a need to develop methods that can balance the need for accuracy with the need for interpretability.

Here, we present an innovative deep-learning method for the classification of TIS cancer cells that addresses some of these challenges. Our approach utilizes a novel neural-network architecture and leverages on the knowledge learned from CNN pre-trained models (Transfer Learning) and on the combination of multiple algorithms (Ensemble Learning) which allows us to obtain relevant performances in the discrimination of TIS cancer cells also on very limited datasets. The dataset consists of 224 three-channel images collected in three different label-free modalities (SRS, TPEF and Transmission) using our multimodal NLO microscope. This approach achieves a classification accuracy of over 90%. We also incorporate the Gradient-weighted Class Activation Mapping (Grad-CAM) visualization approach (Selvaraju et al., 2017) as an interpretability technique to provide insight into the features that contribute to accurate classification. We demonstrate the effectiveness of our system on an experimental dataset of human hepatic cancer cells, in which the TIS phenotype is induced both via drug-based and radiotherapy treatments (Ghislanzoni et al., 2023). We propose a comparison of our novel, unbiased, and automatic approach for the classification of TIS cancer cells over tumoral cells with other competing deep learning architecture specifically developed for this work. We show that the architecture based on both Transfer Learning and Ensemble Learning achieves the best overall performances. To the best of our knowledge, this is the first time that Transfer Learning and Ensemble Learning methods are applied on a dataset of NLO images. Our combined experimental-analytical approach thus holds promise for improving the accuracy and efficiency of TIS detection, ultimately reducing the risk of cancer recurrence using multimodal information processed in a deep-learning framework. It opens the way to a deeper investigation of senescence classification via deep-learning, potentially leading to new insights into the study of senescence as a cause of cancer relapse. We also foster a further study of this tool as a clinical instrument for senescence identification.

## 2 Materials and methods

### 2.1 Input images and dataset

**FIGURE 1:**
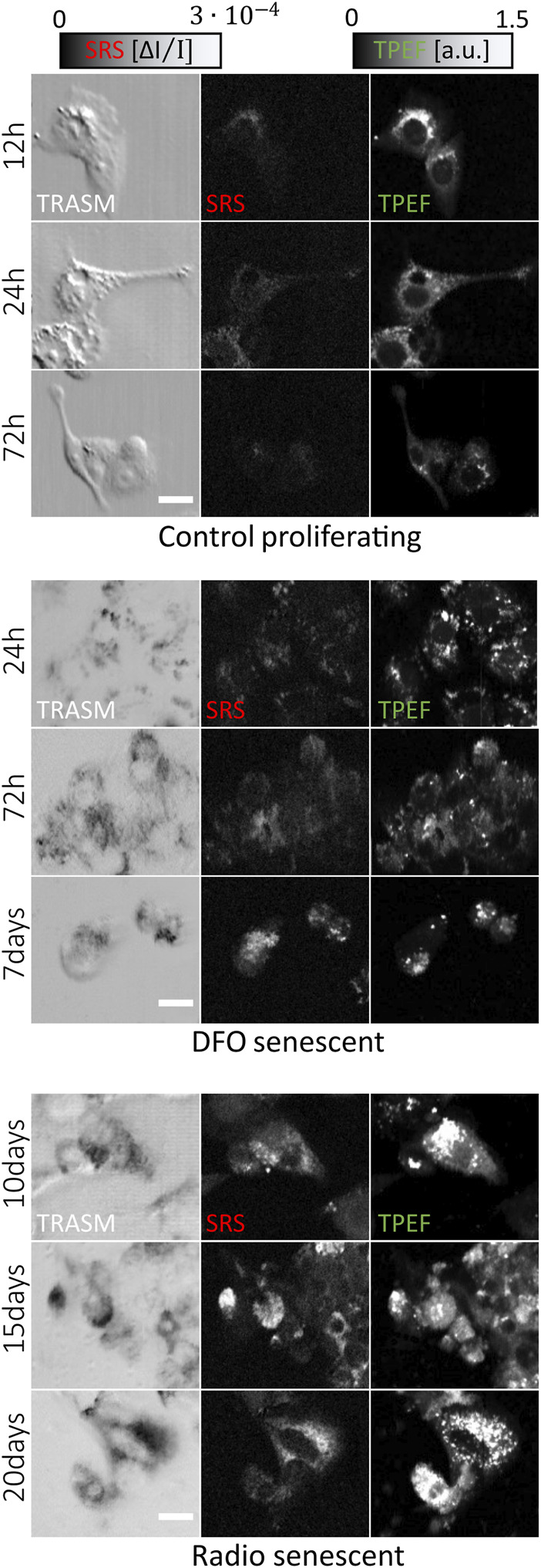
Overview of the multi-channel dataset of HepG2 cells for different treatment and culture conditions. One row represents a typical set of 3 co-registered images that are fed as input to the neural network. Each image is 250 × 300 ${pixels}^{2}$ , acquired from a field of view of 88 × 105 ${\mu m}^{2}$ in size. The scalebar is 20 µm.

Part of the data set was collected in our previous work (Bresci et al., 2023). The new cancer cells populations used for this study were grown in the same conditions and treated to display TIS features using either deferoxamine-based (DFO, MERK) or radiotherapy treatments. Overall, we employ 20 untreated, 25 DFO-treated and 15 irradiated culture plates. Figure 1 presents an extract of the different cell phenotypes used for this study. The new multimodal images are acquired on the same NLO microscope with unchanged experimental protocol and instrumentation, and their ground-truth annotations are assigned by visual inspection. Images are labeled as “Senescent” if they exhibit TIS features, or otherwise labeled as “Control”, based on the markers established previously via NLO microscopy (Bresci et al., 2023).

For the training and evaluation of the neural networks, we acquire a dataset of 224 three-channel images. Out of this dataset, 170 images are used as input for the training of the networks (training dataset), while the remaining 54 images are used for the evaluation of the networks performances and not used in the training process (test dataset). For the training dataset, among the 170 images, 95 are images coming from TIS cells and the remaining 65 are from untreated cancer cells (control cells). For the test dataset, 30 images come from TIS cells and 24 from control ones. A validation dataset is also randomly extracted from the training dataset and used in the training process, to stop it before overfitting. The validation dataset is constituted of 43 images, divided into 24 senescent images and 19 control ones. Every image is composed of three channels, two NLO channels (SRS, TPEF), providing chemical and metabolic information, and the optical transmission channel, providing morphological information. The size of every image is 250 × 300 ${pixel}^{2}$ , covering a field of view of 88 × 105 ${\mu m}^{2}$ , corresponding to a pixel size of 350 × 350 nm^2^. The sampling conditions were determined based on the trade-off between the minimum spatial resolution needed to resolve the smallest biological features of interest and the maximum permitted optical power in order not to alter the sample conditions. Before starting our experimental campaign, we tested different measurement parameters to find the most optimal one for our system. We observed that increasing the sampling rate inevitably caused quick culture medium depletion, significantly reducing the total number of images that could be extracted from one sample, which is a critical feature to create a solid microscopy dataset for deep learning classifiers. In order not to distort the physically acquired signals, before using these images as input of the networks, we only employ a shallow pre-processing step. Therefore, the input images undergo a step of outliers removal using percentile metrics (Liao, Li, and Brooks, 2016) and background noise suppression. Then, the signals from the three channels are normalized and scaled between 0 and 255 to ensure that the pixel values fall within boundaries usable by the pre-trained networks.

Even if the overall dataset is rather large from an experimental point of view (224 fields of view, composed of 250 × 300 spatial and three spectral pixels for a total of more than 50 M values and more than 1000 cells for the whole dataset), this is still fairly small to train a deep-learning network. To solve this issue, we increase the dataset size, applying a data-augmentation step before the networks (Shorten and Khoshgoftaar, 2019). In this step we apply random transformations to the images which do not modify their informative content. These transformations are random rotations, translations, and flipping of the images, where we use a filling mode with zero to handle that after these transformations there are portion of the 250 × 300 pixels images where the original image is not present. We avoid employing cropping and stretching transformations, because these could introduce some unnatural deformations in the positions and concentrations of lipids and mitochondria which could lead to a poor generalization ability of the algorithms. Using the image augmentation step, we obtain 11 transformed images from every single image, bringing our final training dataset to 2040 three-channel images. The image augmentation step is used only to augment the training dataset and not the test one, in order not to introduce data leakage in the evaluation.

### 2.2 Experimental label-free NLO data from cell cultures

We employ a lab-built multimodal NLO scanning microscope to collect morphological, metabolic, and chemical information from HepG2 cells. The microscope has been described in detail in (Crisafi et al., 2018). Briefly, our system is based on a multi-branch Erbium-doped amplified fiber laser that yields a pump beam at 780 nm and a Stokes beam, tunable in the range between 950 and 1050 nm, with 40 MHz repetition rate. The pump beam is modulated with an acousto-optic modulator at 1 MHz. An in-line balanced detection scheme was employed for the SRS measurements, based on what is described in Crisafi et al. The temporal overlap of the two trains of pulses is achieved by operating a manual delay line, positioned on the path of the Stokes beam. The two beams are spatially combined with a dichroic mirror and sent into our homebuilt vertical microscope. The beams are focused via a water-immersion 100X 1.25NA 0.25 mm working distance (WD) objective (C-Apochromat, Carl Zeiss, Germany) and collected by an oil-immersion 40X 1.30NA 0.19 mm WD (CFI Super Fluor, Nikon, Japan) objective. The average laser powers were kept constant on the sample plane at 7.5 mW for the pump and 0.5 mW for the Stokes for all measurements. The Stokes power is limited by the laser source and by the in-line balanced detection scheme, which is fundamental to achieve almost shot-noise-limited performances. Several nonlinear processes can stem from synchronized dual-beam excitation, such as SRS and TPEF. Multispectral detection was performed via a photomultiplier, for the TPEF modality, and a balanced photodiode, serving both for the SRS modality and transmission light modality, on the same field of view. Following two-photon excitation via the 780 nm pump beam, fluorescence signal was epi-detected in the 400–600 nm range using a short-pass filter (FESH0600, Thorlabs), to cover the complete emission spectra of Flavin Adenine Dinucleotide (FAD) and Nicotinamide Adenine Dinucleotide (NADH). These are important coenzymes which act as electron acceptor and donor, respectively, in key metabolic pathways, such as glycolysis, Krebs cycle, and oxidative phosphorylation (Heikal, 2010). Compared to their reduced (FADH_2_) and oxidized (NAD^+^) forms, these molecules are also autofluorescent and represent excellent endogenous sources of optical contrast, offering a way to monitor subtle changes in cellular metabolism (Georgakoudi et al., 2012). Single-channel SRS was detected at 2850 cm^−1^, corresponding to the strong CH_2_ stretching mode of lipids. SRS microscopy at this Raman mode has been proven to provide reliable measurements of lipid droplets in different cell lines, allowing both for visualizing their distribution and quantifying their cellular concentration in a non-invasive, label-free fashion (Gupta et al., 2019). Therefore, the acquisition of these three imaging channels is particularly convenient and efficient for our multimodal NLO system, allowing us to seamlessly obtain a co-registered three-channel image in a single measurement. At the same time, these channels comprise informative but not strongly correlated features, which is extremely advantageous to improve the training process and achieve the best classification performances of a neural network (Toloşi and Lengauer, 2011).

### 2.3 Cell culture, treatment, and sample preparation

HepG2 cells were acquired from the American Type Culture Collection (ATCC, Manassas, VA, United States; ATCC number: HB-8065), and kept in Dulbecco’s modified Eagle’s medium (DMEM) (Gibco), supplemented with 10% fetal bovine serum (FBS) at 37°C and 5% CO2. For NLO analysis, cells were seeded on quartz slides measuring 22 mm × 22 mm × 0.17 mm (Fuzhou Devotop Photonics, China). Each quartz slide was placed in a Ø 6 cm petri dish; for each sample, 320.000 HepG2 cells were suspended in a 1 ml drop of culture medium and plated on a quartz slide inside the petri dish. Then, the sample was placed in an incubator for 2 h. After that, 4 ml of culture medium was added to the petri dish. This step was for cells to only attach and grow on the quartz slides. Before being introduced in the microscopy unit, each square quartz slide was flipped over and sealed on top of a second 25 mm × 50 mm × 0.17 mm quartz slide. TIS features were promoted using either drug-based or radiotherapy treatments. For the former, DFO was employed, diluted in distilled water, reaching a concentration of 100 µM in the culture medium. 24 h after seeding, DFO was introduced in the culture media, and cells were cultured for 12, 24, 72 h, or 7 days. At the end point of treatment, cells were fixed in 4% PFA for 10 min and then put into storage at −4°C. Cells fixed immediately following the administration of DFO are referred to by the temporal control of 0 h. For radiotherapy, 48 h after plating, cells were irradiated at 10 Gy released with gamma-rays from Cs55137 sources of IBL147 biological irradiator (0.65 Gy = min) and maintained at 37°C and 5% CO2. Fresh culture media was added every 96 h. At the end point (5, 10, or 15 days after irradiation), cells were fixed in 4% PFA for 10 min and stored at −4°C.

### 2.4 Model architectures and training

**FIGURE 2:**
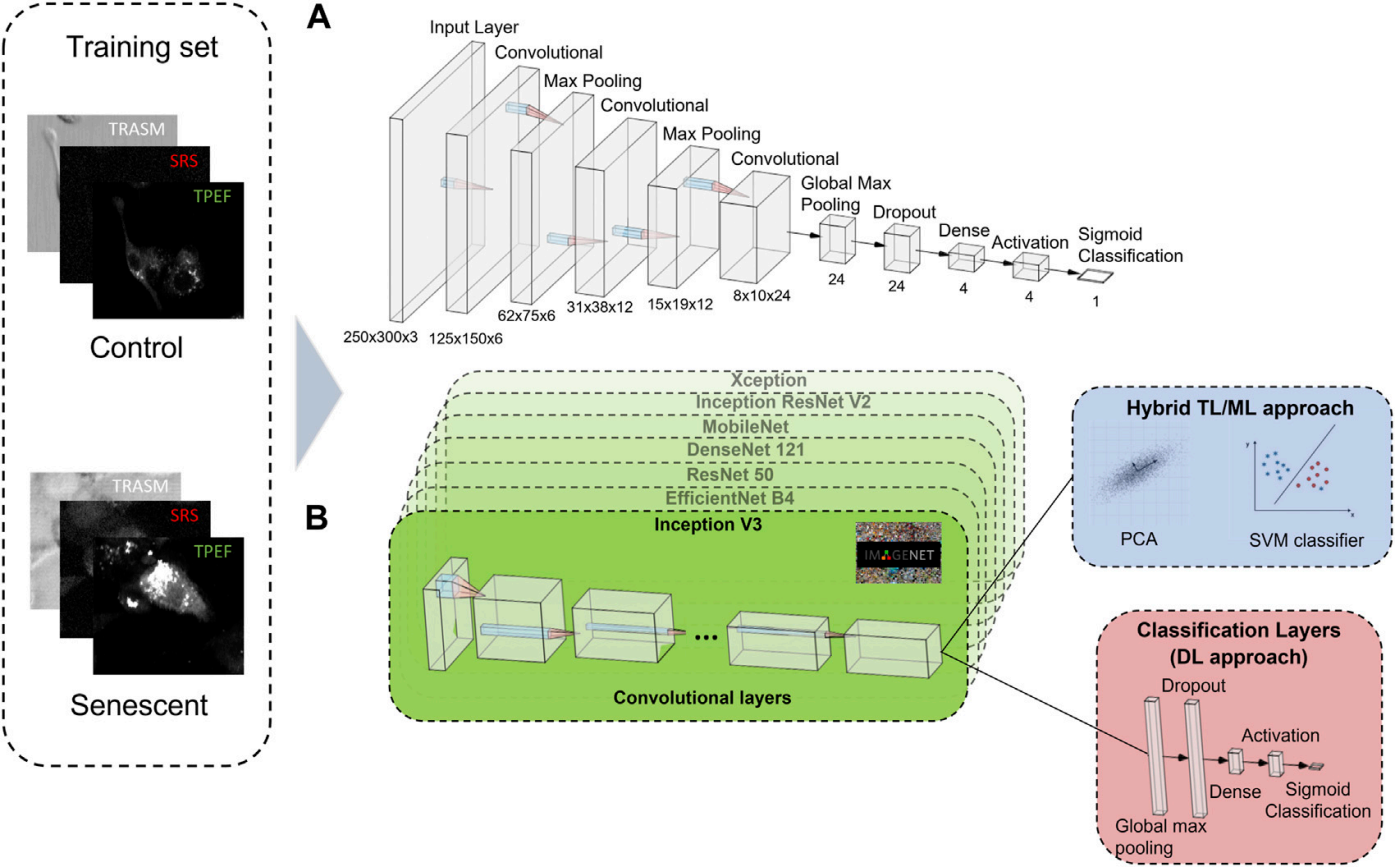
A schematic illustration of the building blocks of the CNN architectures used. **(A)** The CNN from scratch contains three convolutional layers, two 2D max pooling layer, a global max pooling and a dropout layer. The classification task is performed using two fully connected layers of 4 neurons, with a ReLu activation function, and 1 neuron, with a sigmoid activation function. **(B)** The TL approach features seven different pre-trained networks that extract the most relevant information of the three-channel image into a 1D array. The classification task is performed either using a combination of PCA and SVM (ML approach, blue box), or via some classification layers built on top of the pre-trained networks (DL approach, red box). The 3-channel label-free microscopy images in input to the neural networks consist of optical transmission (TRASM), SRS at the 2850 ${cm}^{-1}$ Raman mode of lipids (SRS), and TPEF of the intrinsic NADH and FAD coenzymes (TPEF).

For the classification of cells in untreated tumoral (control) or senescent cells, we first design a CNN classifier from scratch. Our network consists of three convolutional layers whose number of filters are respectively 6, 12, and 24 and with a 3 × 3 kernel size. The aim of the kernel in a CNN is to extract features from the input image by detecting specific patterns such as edges, corners, and other simple shapes (Ajit et al., 2020). In between two consecutive convolutional layers, a 2D max pooling layer is interposed, to reduce the size of the 2D input array by taking the maximum value within a defined window (Zafar et al., 2022). After the last convolutional layer, a global max pooling and a dropout layer are employed (Zafar et al., 2022) (Srivastava et al., 2014). The classification task is then performed using two fully connected layers with respectively 4 neurons, with a ReLu activation function (Fukushima, 1975), and 1 neuron, with a sigmoid activation function (Narayan, 1997). Figure 2A shows all the layers used for building the neural network from scratch and their corresponding sizes.

Besides that, since we dispose of a small number of instances for the dataset, we also develop architectures based on the Transfer Learning (TL) approach. TL is a method that uses complex CNNs that were pre-trained on a large, labelled image database (such as ImageNet (Deng et al., 2009)) to perform classification tasks (Weiss et al., 2016). This method is particularly convenient in cases in which the available datasets are constituted by a reduced number of instances. Indeed, TL takes advantage of the knowledge acquired in the pre-training of a complex neural network on an exceptionally large dataset to perform classification on a different, previously unseen dataset, after a fine tuning to adapt the learned parameters to the specific network to fulfill the task. This is possible because the lower-level features learned from pre-training on large datasets (like edges, geometric shapes, etc.) are often generalizable to a wide range of tasks, allowing the model to extract relevant features from the new data with less training data. Some pre-trained neural networks are available open source. In this work, we employ the following seven different pre-trained open-source networks: Inception V3 (Szegedy et al., 2016), EfficientNet-B4 (Tan and Le, 2019), DenseNet 121 (G. Huang et al., 2017), ResNet50 (Szegedy et al., 2017), MobileNet (Howard et al., 2017), Xception (Chollet, 2017), Inception ResNet V2 (Szegedy et al., 2017). All these pre-trained networks are trained on the same ImageNet database, which is a database of RGB images, and using as input the three RGB channels of the images. These architectures are particularly convenient for our task, since our NLO cell-images also consists of three channels (SRS, TPEF and Transmission).

For the first architecture using TL, we employ a hybrid TL and Machine Learning (ML) approach, that utilizes all the seven pre-trained networks presented before and is shown in Figure 2B (blue box). The algorithm consisted of two parts. In the initial part, an “off-the-shelf” pre-trained network (without network retraining) is used (Zhuang et al., 2021). This network transforms the input image into a 1D vector, the size of which is dependent on the chosen pre-trained network. Whereas, in the second part, the classification is performed first using a Principal Component Analysis (PCA) (Jolliffe et al., 2016) to reduce the dimensionality of the 1D vector and then a Support Vector Machine (SVM) (Hearst et al., 1998) with a polynomial kernel for the actual classification. PCA is a widely used dimensionality reduction technique that transforms high-dimensional data into a lower-dimensional space by identifying the orthogonal directions of maximum variance, while SVM is a powerful ML algorithm that identifies a hyperplane in a high-dimensional space to effectively separate different classes of data points by maximizing the distance between them. The best parameters of the SVM are learned autonomously by the system using the Cross-Validation method (Yadav, 2016). As each of the seven different pre-trained networks provides an independent classification, the final label assigned to an image in the test set is obtained through a hard voting scheme, where the class with the highest number of votes is assigned as the label.

Successively, always in the TL framework, we design a more sophisticated method, entirely based on deep-learning, which is sketched in Figure 2B (red box). In this case, we build a small and dense neural network on top of the pre-trained networks, composed by a 2D global max pooling, followed by a dropout layer (dropout rate = 0.8), a 4-neuron dense layer with ReLu activation function and a dense 1-neuron layer with sigmoid activation function, thus providing as an output the probability that the input image effectively belongs to the senescent class. The overall network undergoes a double training process. At the beginning, the pre-trained network is used in the “off-the-shelf” manner, where only the weights of the final dense layers are trained. Subsequently, the parameters of the pre-trained network are also fine-tuned, to better adapt them to the multimodal NLO microscopy images, together with a further fine-tuning also of the final dense layers weights. Indeed, because the pre-trained networks were trained on RGB images, which are structurally different from the NLO cell-images used for our TIS classification, the pre-trained weights need a fine-tuning step. However, this second part of the training could lead to a strong overfitting, due to the small size of the dataset and the large number of parameters involved, therefore this fine-tuning is performed with a very small learning rate (2 x $10^{-5}$ ), which allows us to obtain better performances as it will be shown in the Results section. Eventually, this approach results in seven TL classifiers, whose performances vary depending on the specific pre-trained network employed.

The last approach we employ is based on the Ensemble Learning (EL) technique (Dietterich, 2000). EL is a strategy that aims to enhance the performance and the stability of a single model by combining multiple simple models (weak learners). This is achieved by promoting significant diversity among the models, which can be accomplished by training the individual models on a subset of features or a portion of the entire dataset. In the realm of machine learning, Random Forest is an example of EL based on simpler Decision Tree (Breiman, 2001). The predictions of the simpler models can be combined in a weighted scheme or non-weighted voting scheme, which is the one used in this work. Namely, in a non-weighted voting scheme with n learners, every learner $l_{i}$ predicts a probability $p_{ij}$ for every class $c_{j}$ . These probabilities are then equally averaged and the predicted class of the whole architecture is the one with higher probability, therefore $Predicted\;Class={\mathrm{argmax}}_{j}\left(\frac{1}{n}\sum_{i=1}^{n}p_{ij}\right)$ .

**FIGURE 3:**
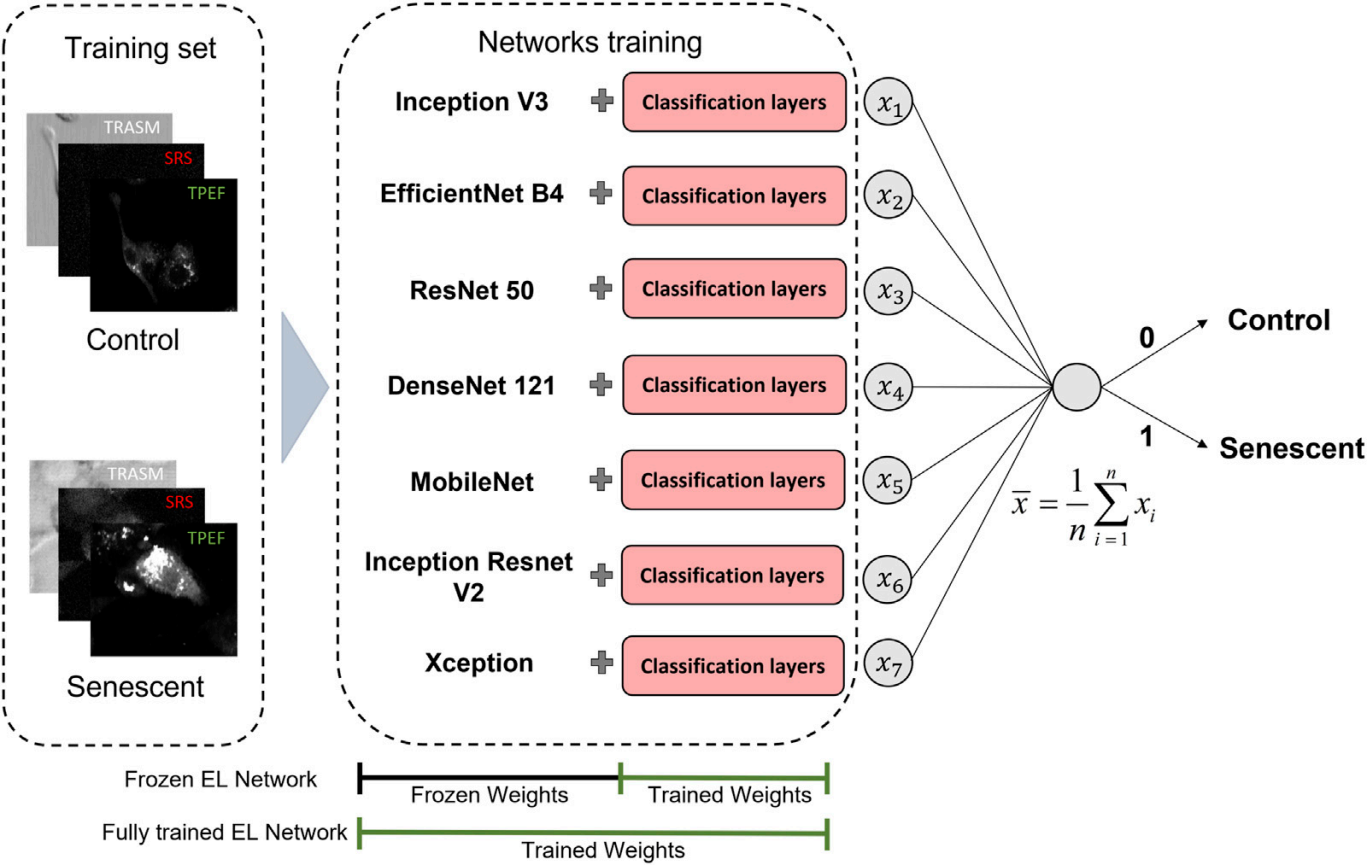
Workflow of the proposed EL framework for the classification of TIS (Senescent) and proliferating (Control) cancer cells. We investigate two different TL scenarios, in which the classification probability is given by the average of the probabilities predicted employing seven different pretrained networks. In the first actualization, the weights of the pre-trained networks are left untouched, while in the final model, both the weights of the classification layers and the pre-trained networks are fine-tuned. The 3-channel label-free microscopy images in input to the neural networks consist of optical transmission (TRASM), SRS at the 2850 ${cm}^{-1}$ Raman mode of lipids (SRS), and TPEF of the intrinsic NADH and FAD coenzymes (TPEF).

In our case, the ensemble is composed of the seven pre-trained networks cited before, which are considered as the weak learners, to which the fully connected classification layers are added, as already shown in the red box of Figure 2B. These networks are first used in the off-the-shelf manner (frozen EL network) and then applying fine-tuning also to the pre-trained weights (fully trained EL network). In Figure 3 we give a visual representation of the ensemble technique here described, indicating in both cases which portion of the network was affected by the training. The cost function employed for the individual training of the networks is the binary cross-entropy

$$Cross\;Entropy=-\frac{1}{N}\cdot \sum_{i=1}^{n}y_{i}\log\left(p_{i}\right)+\left(1-y_{i}\right)\log\left(1-p_{i}\right)$$ (1)

where $y_{i}$ is the ground truth label of image i and $p_{i}$ is its probability of being senescent extracted from the network at each epoch (Zhang et al., 2018). We adopt a L1 and L2 penalty scheme with a 0.01 penalty coefficient for the 4-neuron dense layer, a batch size of 20 images, a maximum of 200 epochs, a learning rate of 5 x $10^{-4}$ for the training of the dense layers and a learning rate of 2 x $10^{-5}$ for the pre-trained weights fine-tuning. Moreover, in the training process, to avoid strong overfitting, we use an early stopping training with a patience of 20 epochs, which is based on the use of a validation dataset randomly extracted from the training dataset and aimed to stop the training if the binary cross-entropy on the validation set does not decrease of at least 0.005 over a patience of 20 consecutive epochs. Indeed, overfitting is a fundamental issue in ML which occurs when a model is trained too much on the training dataset, leading to poor performance on an unseen test dataset (Ying, 2019). To introduce randomness in this scheme and further improve the power of the ensemble, we generate a different augmented training dataset for each of the seven pre-trained networks, because this leads to a higher variability among the seven networks, leading to a higher generalization ability of the model (Dietterich, 2000). The predicted probabilities coming out from the seven networks are then simply averaged in a non-weighted voting scheme and the ultimate classification is based on the class with larger averaged probability.

All the neural networks described are implemented in Python 3.8.8 using Tensorflow 2.5.0 and Keras 2.5.0. The training of the networks was performed using an NVIDIA GeForce RTX 3090 GPU. This allows us to have a training time of around 2.5 h for the fully trained EL network and a prediction time for all the 54 images in the test set of just 1.3 s, namely 24 ms per image. Moreover, 24 ms is less than the acquisition time for every image and therefore this tool could also support real time classification. The program code used in this work is available for use and re-use under an open-source license and can be accessed via GitHub (https://github.com/salvasorrentino/deep_learning_senescence).

### 2.5 Metrics for networks performance evaluation

To evaluate our different classification architectures, we use the following metrics for the different algorithms trained: accuracy, precision, recall, F1-score, and AUC (J. Huang and Ling, 2005). Accuracy is defined as the percentage of instances in the test set properly classified by the algorithms; precision is the ratio of senescent cells properly classified over whole number of senescent cells predicted from the test set; recall is the ratio of senescent cells properly classified over the whole number of senescent cells in the test set, F1-score is a harmonic average of precision and recall. The definition of these metrics in terms of True Positive (TP), True Negative (TN), False Positive (FP) and False Negative (FN) is presented in Table 1. AUC is defined as the Area under the Receiver Operating Characteristic (ROC) curve, where the ROC curve is a plot showing the diagnostic ability of a binary classifier as its discrimination threshold is changed.

**TABLE 1:** The performance metrics used to compare the different classifiers. TP is an outcome where the model correctly predicts the senescent class, whereas TN is an outcome where the model correctly predicts the control class. Conversely, FP is an outcome where the model incorrectly predicts the senescent class, and FN is an outcome where the model incorrectly predicts the control class.

Metrics	Formula
Accuracy	(TP+TN)/(TP+TN+FP+FN)
Precision	$TP/\left(TP+FP\right)$
Recall	$TP/\left(TP+FN\right)$
F1−score	$2TP/\left(2TP+FP+FN\right)$

## 3 Results

**FIGURE 4:**
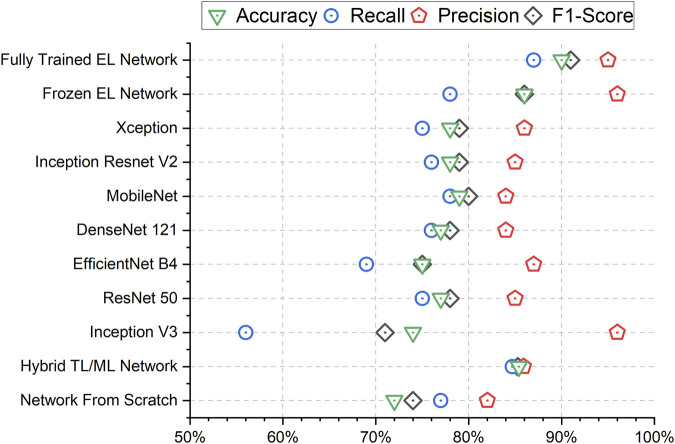
Mean Accuracy, F1-Score, Precision, and Recall results for all the trained networks. The Fully Trained EL Network performs consistently well in every metric. The higher precision of Inception V3 and the Frozen EL Network are due to a low number of cells classified as Senescent as it is clear from the very low Recall.

The results in terms of accuracy, precision, recall and F1-score for the different approaches are presented in Figure 4. To obtain metrics that are independent of chance-based correct classifications during a single training, we calculate the mean and standard deviation of the metrics across nine different training runs of the networks, thereby providing a measure of uncertainty over the trainings. Overall, the fully trained EL network has demonstrated superior performances over the competing methods both in terms of mean values and standard deviations. The fully trained EL network is the approach with the highest performances in terms of accuracy (90.1%), recall (87.4%) and F1-score (90.8%). Among the competitors, Inception V3 achieves the highest precision (95.7%), but this result is due to the imbalance between the predictions as senescence or control of this network, as confirmed by the poor recall (55.9%), meaning that Inception V3 fails to recognize many senescent images. Looking at the seven networks which compose the ensemble, we observe that the accuracy ranges from 74.1% (Inception V3) to 78.6% (MobileNet), which is lower than the values obtained both by the fully trained EL network and the frozen EL network (85.8%). Finally, while the hybrid TL/ML approach (85.4%) and the network from scratch (71.5%) do not reach competitive performances for the accuracy, the former interestingly scores better and more consistently than most of the other models, and comparable to the frozen EL network.

**FIGURE 5:**
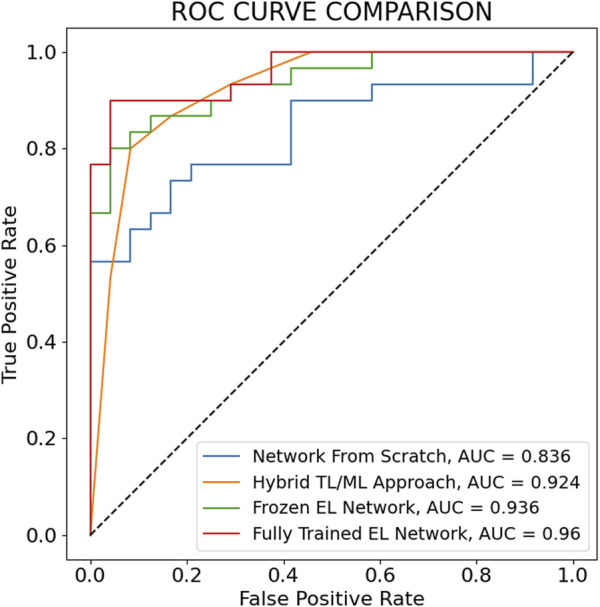
ROC curves corresponding to the maximum AUC over the 9 training steps for the main architectures considered for the classification of Control *versus* Senescent cells. All the ROC curves except the one for the TL/ML approach are drawn calculating the True Positive Rate and False Positive Rate adjusting the classification probability threshold. In the TL/ML approach the True Positive Rate and False Positive Rate are calculated using as threshold the number of pre-trained networks (out of seven) predicting senescence through the final SVM level.

The maximum AUC over the nine training steps and the corresponding ROC curves for the fully trained EL network, the frozen EL network, the hybrid TL/ML approach, and the neural network trained from scratch are reported in Figure 5. Also in this case, the fully trained EL network performs best, with the highest AUC score (0.960), larger than the one of the frozen EL network (0.936), while the hybrid TL/ML approach presents an AUC of 0.924, which confirms the slightly lower performances compared with the EL approaches. Among the individual pre-trained networks, ResNet 50 is the one with the best score (0.919, not shown in Figure 5). Table 2 summarizes all the performances in terms of average accuracy, precision, recall, F1-score, and maximum AUC over the nine training steps for each presented approach.

**TABLE 2:** Performances of the classifiers across nine different training runs, measured in terms of mean and standard deviation (SD) for every metric. For the AUC is reported the maximum value through the nine training steps. All values are given in percentages.

Model	Accuracy ± SD	Precision ± SD	Recall ± SD	F1-Score ± SD	Max AUC
Fully Trained EL Network	90.1 ± 1.2	94.5 ± 2.2	87.4 ± 2.6	90.8 ± 1.2	96.0
Inception V3	74.1 ± 2.6	95.7 ± 3.4	55.9 ± 4.7	70.5 ± 3.7	89.4
ResNet 50	77.3 ± 3.9	84.8 ± 8.5	74.8 ± 14.0	77.9 ± 6.2	91.9
EfficientNet B4	75.3 ± 2.8	86.6 ± 9.7	68.9 ± 14.4	75.0 ± 5.5	83.6
DenseNet 121	77.2 ± 3.8	83.8 ± 8.1	75.6 ± 12.6	78.2 ± 5.5	90.6
MobileNet	78.6 ± 4.6	84.0 ± 7.5	77.9 ± 12.4	79.7 ± 6.0	91.4
Inception Resnet V2	78.4 ± 4.4	85.4 ± 7.8	75.8 ± 12.6	79.1 ± 5.9	86.9
Xception	78.4 ± 4.2	85.9 ± 7.5	75.0 ± 12.1	79.0 ± 5.7	86.1
Frozen EL Network	85.8 ± 2.9	95.8 ± 2.8	77.9 ± 5.9	85.8 ± 3.3	93.6
Hybrid TL/ML Network	85.4 ± 3.1	85.9 ± 2.9	84.7 ± 3.3	85.3 ± 3.1	92.4
Network From Scratch	71.5 ± 9.4	81.5 ± 16.9	76.5 ± 23.4	73.7 ± 10.7	83.6

**FIGURE 6:**
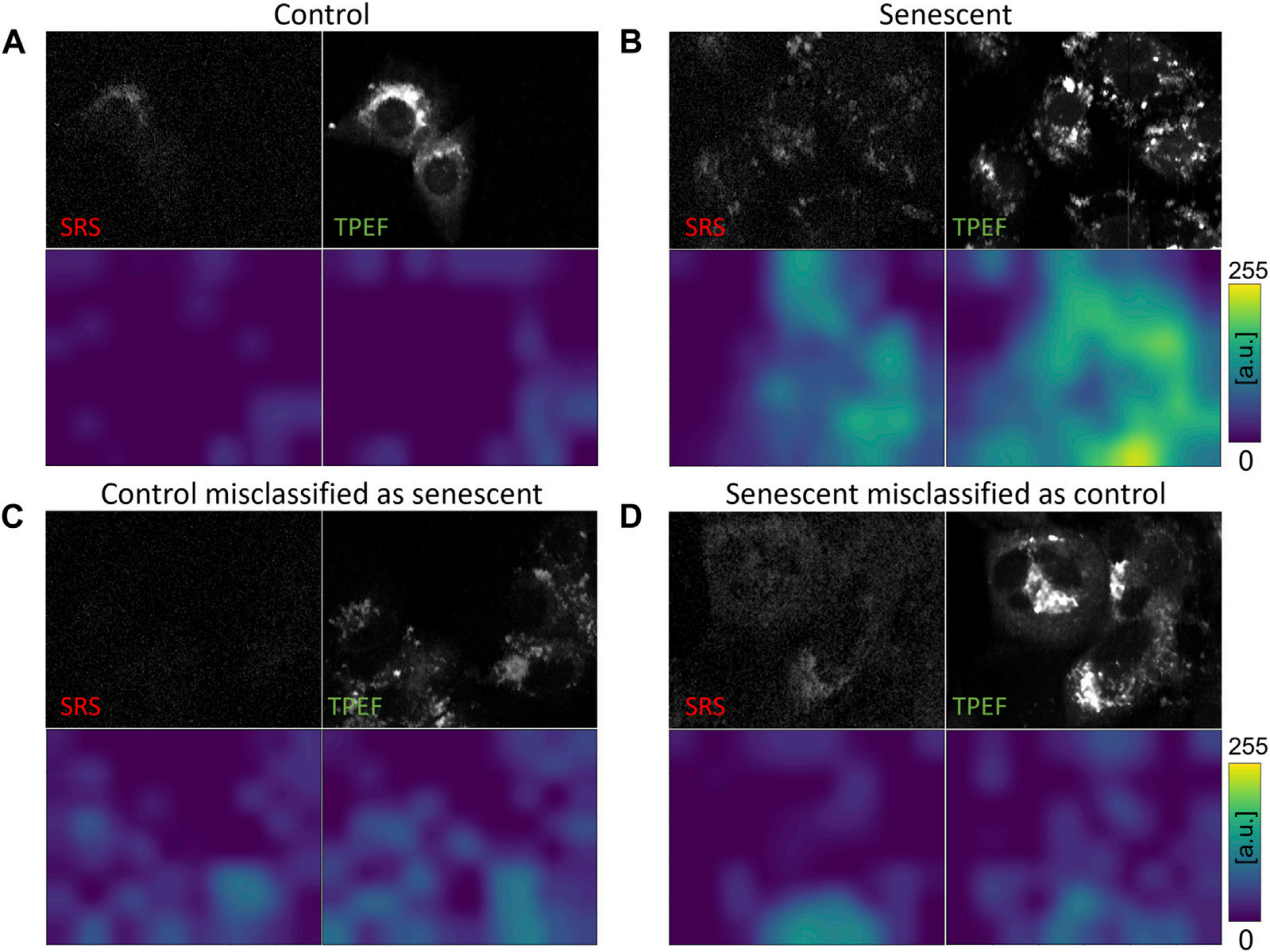
Grad-CAM images relative to SRS and TPEF images of control and senescent cells. For each field of view, the NLO channels (upper row) and the corresponding generated intensity grad-CAMS images (lower row) are presented. Images relative to: **(A)** a Control cell correctly classified by the network; **(B)** a Senescent cell correctly classified by the network; **(C)** a Control cell misclassified as Senescent by the network; **(D)** a Senescent cell misclassified as Control by the network.

In order to substantiate the efficacy of incorporating images encompassing all three channels (SRS, TPEF, Transmission) as input for neural networks, we also conducted training on the fully trained EL network using each of the three feasible two-channel combinations, namely TPEF-Transmission, SRS-Transmission, and SRS-TPEF. Remarkably, these combinations achieved accuracies of 85.8%, 87.2%, and 87.4%, respectively. Detailed outcomes are presented in the Supplementary Material. Even though these results fall short of the 90.1% accuracy obtained by the fully trained EL network with a 3-channel input, they prove the value of using combined information from SRS, TPEF, and Transmission. Indeed, learning from the joint distribution present in all three channels, the network acquires the capability of enhancing the prediction performance on the test set. In addition to the comparison of metrics, for the evaluation of our different classification architectures, we adapt the Grad-CAM visualization approach (Selvaraju et al., 2017) to the fully trained EL network. Grad-CAM is a technique which produces a visual representation of the criteria used by CNN-based models to perform a given classification task, increasing its interpretability. The Grad-CAM approach uses the gradients of any possible label in the CNN network, flowing into the final convolutional layer, to produce a coarse localization map which highlights the most significant regions in the image that contribute to the network prediction (Selvaraju et al., 2017). In this work, we use the Grad-CAM maps to visualize how the fully trained EL network is learning, namely which are the key features inside the image leading to a particular label (senescent or control) in the classification process. We can use this information to understand the possible reasons for the misclassification of some images in the test set. Since our classifier outputs 1 for the senescent label and 0 for the control label, it implies that the brighter the pixels in the map, the higher is their importance for the classification of the image as senescent. Conversely, we expect that a cell classified as proliferating (i.e., control) features dark pixels in the Grad-CAM map. We derive these maps both for the SRS channel and the TPEF channel, which are the two main channels used in our previous work (Bresci et al., 2023) to extract statistical indicators of senescence. Hence, in Figure 6 we present eight Grad-CAM maps, with their corresponding SRS and TPEF signals, both for correctly classified and misclassified control and senescent images. Despite the limited spatial resolution of the approach, due to the 6 × 8 pixels size of the extracted maps that are then upscaled to the image size (250 × 300 pixels) via linear interpolation, the maps confirm that the network is effectively learning the primary cellular features of the images that are responsible for classification. The 8 × 6 pixels size of the maps comes directly from the size of the last convolutional layer and is a consequence of the squeezing effect induced by the kernel in this layer.

## 4 Discussion

The simplest approach that we use as a baseline for our study is represented by the training of a CNN network from scratch. This approach leads to poor results in terms both of accuracy (71.5%) and AUC (0.836) and this is due to the small number of available images for the training of a neural network from scratch. To overcome this limitation, TL approaches using seven different pre-trained networks are employed. The hybrid TL/ML architecture goes in this direction and allows us to obtain a higher result in terms of accuracy (85.4%) and AUC (0.924). A more complex architecture is obtained adding fully connected layers on top of the pre-trained networks. Looking at Table 2, the performances in terms of accuracy and maximum AUC of each of the individual pre-trained networks do not surpass the ones of the hybrid TL/ML approach. This is because the fully connected layers added to the pre-trained networks necessitate of a great number of parameters to be trained compared with the TL/ML approach, and this likely leads to an overfitting, which is the reason for the poorer performances. However, when the seven pre-trained networks are considered together to constitute the ensemble in the frozen EL network, we report a slight improvement in performances for accuracy (85.8%), F1-score (85.8%) and AUC (0.936) with respect to the TL/ML approach. In the fully trained EL approach, following a fine-tuning of the pre-trained networks parameters, we observe an additional improvement in performance, which leads to an accuracy of 90.1%, an F1-score of 90.8% and an AUC of 0.960. The fully trained EL network is the one with both the best overall performances for the classification of senescent and control cells and the one with the smallest standard deviation in all the metrics, which corroborates the idea of an increase not only in performances but also in stability of the EL techniques. This is clearer also comparing the metrics of the fully trained EL network with the individual pre-trained networks which compose the ensemble, where we notice that the accuracy of these networks is in average 13% less than the accuracy of the fully trained EL network, which proves the significant increase in performances of this approach. Moreover, the fully trained EL network presents an accuracy 4.3% higher and a standard deviation 1.7% smaller with respect to the frozen EL network. This means that the fine tuning of the parameters of the pre-trained networks does not increase the model overfitting, because otherwise we would observe a performance deterioration, but allows the algorithm to adapt it better to the non-RGB images in the training dataset, which increases the generalization capabilities of our network. This is a relevant result, because prove the possibility of applying usual TL trained on common RGB images also for tasks which involve non-RGB images, after a slight fine-tuning of the pre-trained networks weights.

Finally, we examine the evidence presented in the Grad-CAM maps displayed in Figure 6, which are derived from the fully trained EL network. The maps are generated based on the gradient extracted from the network. As shown in Figure 6B, cells classified as senescent exhibit strong activation in the maps for both SRS and TPEF images. This activation is spatially colocalized with the bright cellular regions of intense SRS and TPEF signals in Figure 6B, which can be associated respectively to lipids aggregation and mitochondrial network aggregation, previously identified as optical markers for TIS using statistical tools (Bresci et al., 2023). Conversely, as depicted in Figure 6A for a properly classified control cell, the Grad-CAM maps display negligible activation. This observation is coherent with our previous discussion, since it means that no pixels caused the network to activate towards the prediction of the senescence class. Furthermore, we inspect the Grad-CAM maps for the cases of image misclassification, thus gaining useful insights for a better comprehension of the causes leading to the wrong outcome. Figure 6C shows a control image misclassified as a senescent one. Typically, in control cells, mitochondria form a network of connections that spread over the whole cytoplasm and surrounds the nuclei. Consequently, the TPEF signal from these cells is quite uniform and the nuclear regions are clearly visible. Compared to Figure 6A then, the TPEF signal here appears localized in unusual, isolated bright spots and the nuclei are not clearly distinguishable. As such, the associated Grad-CAM map presents high value pixels roughly colocalized with the regions of intense TPEF signals, which were probably mistaken as features of mitochondrial aggregation. On the other hand, Figure 6D reports the case of senescent cells misclassified as control ones. While both the SRS and TPEF images present intense signals, these are widely distributed over the whole cellular area, similarly to Figure 6A, making it difficult to assign the correct class. Indeed, cellular response to therapy changes over treatment time, as evident from Figure 1, so that early-stage TIS cells can display optical features that closely resemble those of control cells, making them harder to recognize.

The reported results demonstrate the usefulness of applying networks pre-trained on standard RGB images for the classification of non-RGB images and this is thanks to the ability of the convolutional layers to extract universal features from the images. Moreover, we prove that the use of ensemble network combined with TL leads to a strong improvement in the ability of the algorithm to distinguish between the two classes of cells. The present study builds upon previous work on the combined use of TL and EL for image classification in cervical histopathology (Zheng et al., 2022). However, our approach differs in that we employed a greater number of pre-trained networks to constitute the ensemble and extended the application of both the ensemble and the transfer-learning technique, pre-trained on RGB images, to classify non-RGB images of cells. Similarly to a previous work on the application of TL to colon cancer classification using confocal microscopy (Gessert et al., 2019), we assess the performance of multiple pre-trained networks on non-RGB images and compare them with other viable models. However, rather than selecting the top-performing algorithm based on our evaluations, we also leverage these comparisons to converge to a final solution that integrates the strengths of the different networks via the EL approach.

In this work we investigate the performances of several deep-learning algorithms to find the best classifier for the discrimination between TIS and control cancer cells via NLO microscopy measurements. We develop an algorithm based on TL and EL which achieves more than 90% accuracy and F1-score. In addition, we prove the strong increase in performances and network stability obtained by EL. Compared with our previous work (Bresci et al., 2023), our classification algorithm does not rely on statistics to distinguish between the two types of cells and, above all, provides an automatic and unbiased system to perform the classification task. In addition, thanks to the multimodal optical modalities at our disposal, our approach combines morphological, chemical, and metabolic information, increasing the predictive power of the network. The lack of labelled images is overcome using data augmentation, TL, and EL techniques. This demonstrates that it is possible to build a robust and accurate classifier for non-RGB images, also starting from a small dataset by applying more sophisticated models. Despite these advantages, our fully trained EL network incurs a higher computational cost than neural networks trained from scratch or using hybrid TL/ML approaches, and this cost scales with the number of training images. Our methodology addresses a binary classification problem. In this respect, we envisage future research directions focusing on the development of a multiclass classifier capable of not only distinguishing between TIS and control cells, but also explaining how cells may progress through stages of cellular senescence stage of the cells. The achievement of this goal will require a larger dataset with additional label classes corresponding to the different time-points following the multi-step progression of senescence. We anticipate that an advancement in the Grad-CAM methodology, enabling higher resolution maps and thereby enhancing our understanding of how the network is learning, could enable the discovery of novel features in the SRS and TPEF signals that would indicate the presence of TIS cells and identify their time stage. Moreover, the classification power of the system could be greatly increased with the addition of further label-free NLO channels to the network input, such as SRS images targeting the Raman signatures of proteins and nuclei, which proved to be effective chemical markers of senescence (Oh et al., 2022). Our novel pre-trained algorithm, capable of accurately identifying TIS cells in human liver cancer cells, presents potential applicability also to cancer cells different from HepG2. Indeed, by fine-tuning the weights of the neural network using datasets from different cell lines, our approach could potentially be studied also on human patients’ samples to offer a fully automated tool for detecting TIS cells in pre-clinical screenings and, ultimately, support clinical diagnosis. The network predictions could provide fast, independent, and unbiased advice, complementing expert evaluation. Furthermore, the Grad-CAM maps integration in the network enables a deeper understanding of the network decision-making process, enhancing the reliability of our tool. Indeed, an end-user lacking technical knowledge about the neural network could use the Grad-CAM maps to execute its due diligence in checking the robustness of the predictions. Notably, our approach allows ranking of cells based on the probability of TIS presence, enabling prioritization of further analysis on images with probability close to the decision threshold of 0.5, thereby it could be studied also for optimizing expert diagnosis efficiency.

## 5 Conclusion

The results of our comparisons show that the most complex deep-learning architecture, the fully trained EL approach using an ensemble of fine-tuned pre-trained networks, provides the best performance in terms of accuracy, AUC and F1-score for the senescence classification task. Moreover, the use of ensemble learning further improves the results and increases the robustness of the classification. In conclusion, we demonstrate that deep transfer learning models, together with ensemble techniques, can effectively be employed for the classification of senescent cells also starting from non-RGB images, namely NLO microscopy signals like SRS and TPEF, opening the way to a larger use of these techniques for automatic classification of non-standard images. We also employ a method for the interpretability of the decision process made by the networks, using Grad-CAM maps, which proves an agreement between the spatial features identified by the networks as the prominent to distinguish senescent and proliferating cells and previous results obtained by Bresci et al., which evidence the characterizing traits for senescence. Adapting this method to have a larger map resolution could shed new light on the understanding of which features are the main markers to discover cell senescence in human cells. We also believe that this unbiased and automatic approach for senescence classification could be tested in the pre-clinical and clinical diagnosis of human cells senescence, becoming a valuable tool employed in senescence detection.

## Data Availability

The datasets presented in this study can be found in online repositories. The names of the repository/repositories and accession number(s) can be found below: https://zenodo.org/record/7946845#.ZG0vfn1BxPZ.
