# Supplementary material for: Deep ensemble learning and transfer learning methods for classification of senescent cells from nonlinear optical microscopy images
Source: Front Chem. 2023 Jun 23;11:1213981. doi: 10.3389/fchem.2023.1213981 (PMC10326547; doi:10.3389/fchem.2023.1213981)
Supplement: Supplementary file 1 [file Table1.DOCX]

Supplementary Material

**Deep Ensemble Learning and Transfer Learning Methods for**

**Classification of Senescent Cells from Nonlinear Optical Microscopy Images**

Salvatore Sorrentino^1^†, Francesco Manetti^1^†, Arianna Bresci^1^, Federico Vernuccio^1^, Chiara Ceconello^1^, Silvia Ghislanzoni ^2^, Italia Bongarzone ^2^, Renzo Vanna^3^, Giulio Cerullo^1,3^, Dario Polli^1,3*^

*** Correspondence:** Dario Polli: dario.polli@polimi.it

# Supplementary Data

To corroborate the usefulness of employing all the 3-channels (SRS, TPEF, Transmission) as input to the neural networks, we also train the fully EL network using all the three possible 2-channels combinations, namely TPEF-Transmission, SRS-Transmission and SRS-TPEF. In order to adapt the pretrained networks to receive in input 2-channels images, instead of the usual 3-channels ones, we add a 3 filters-2D convolutional layer with kernel size 1x1 between the 2-channels input images and the first layer of the pretrained networks. The performances of the networks trained with 2-channels images are evaluated using the same test dataset used for the comparisons of all the networks presented in the main text. In the Supplementary Table 1 all the evaluation metrics for these networks are shown.

# Supplementary Figures and Tables

| **Model** | **Accuracy** ± **SD** | **Precision** ± **SD** | **Recall** ± **SD** | **F1-Score** ± **SD** | **Max AUC** |
| --- | --- | --- | --- | --- | --- |
| Fully Trained EL Network using only TPEF and Transmission channels | 85.8 ± 3.7 | 84.2 ± 4.0 | 91.8 ± 4.5 | 87.8 ± 3.1 | 90.8 |
| Fully Trained EL Network using only SRS and Transmission channels | 87.2 ± 2.7 | 92.6 ± 3.5 | 84.1 ± 6.4 | 87.9 ± 2.9 | 95.4 |
| Fully Trained EL Network using only SRS and TPEF channels | 87.4 ± 2.4 | 88.0 ± 5.7 | 90.4 ± 4.0 | 88.9 ± 1.7 | 94.7 |

**Supplementary Table 1**. Calculated metrics on the test set, with their corresponding Standard Deviation (SD), for the three 2-channels combinations (TPEF-Transmission, SRS-Transmission and SRS-TPEF)
